# Impact of health warning labels and calorie labels on selection and purchasing of alcoholic and non‐alcoholic drinks: A randomized controlled trial

**DOI:** 10.1111/add.16288

**Published:** 2023-08-01

**Authors:** Natasha Clarke, Jennifer Ferrar, Emily Pechey, Minna Ventsel, Mark A. Pilling, Marcus R. Munafò, Theresa M. Marteau, Gareth J. Hollands

**Affiliations:** ^1^ Behaviour and Health Research Unit, Department of Public Health and Primary Care University of Cambridge Cambridge UK; ^2^ School of Sciences Bath Spa University Bath UK; ^3^ School of Psychological Science, Tobacco and Alcohol Research Group University of Bristol Bristol UK; ^4^ EPPI Centre, UCL Social Research Institute University College London London UK

**Keywords:** Alcohol, calorie label, choice architecture, health warning label, purchasing, selection

## Abstract

**Aims:**

To estimate the impact on selection and actual purchasing of (a) health warning labels (text‐only and image‐and‐text) on alcoholic drinks and (b) calorie labels on alcoholic and non‐alcoholic drinks.

**Design:**

Parallel‐groups randomised controlled trial.

**Setting:**

Drinks were selected in a simulated online supermarket, before being purchased in an actual online supermarket.

**Participants:**

Adults in England and Wales who regularly consumed and purchased beer or wine online (*n* = 651). Six hundred and eight participants completed the study and were included in the primary analysis.

**Interventions:**

Participants were randomized to one of six groups in a between‐subjects three [health warning labels (HWLs) (i): image‐and‐text HWL; (ii) text‐only HWL; (iii) no HWL] × 2 (calorie labels: present versus absent) factorial design (*n* per group 103–113).

**Measurements:**

The primary outcome measure was the number of alcohol units selected (with intention to purchase); secondary outcomes included alcohol units purchased and calories selected and purchased. There was no time limit for selection. For purchasing, participants were directed to purchase their drinks immediately (although they were allowed up to 2 weeks to do so).

**Findings:**

There was no evidence of main effects for either (a) HWLs or (b) calorie labels on the number of alcohol units selected (HWLs: *F*_(2,599)_ = 0.406, *P* = 0.666; calorie labels: *F*_(1,599)_ = 0.002, *P* = 0.961). There was also no evidence of an interaction between HWLs and calorie labels, and no evidence of an overall difference on any secondary outcomes. In pre‐specified subgroup analyses comparing the ‘calorie label only’ group (*n* = 101) with the ‘no label’ group (*n* = 104) there was no evidence that calorie labels reduced the number of calories selected (unadjusted means: 1913 calories versus 2203, *P* = 0.643). Among the 75% of participants who went on to purchase drinks, those in the ‘calorie label only’ group (*n* = 74) purchased fewer calories than those in the ‘no label’ group (*n* = 79) (unadjusted means: 1532 versus 2090, *P* = 0.028).

**Conclusions:**

There was no evidence that health warning labels reduced the number of alcohol units selected or purchased in an online retail context. There was some evidence suggesting that calorie labels on alcoholic and non‐alcoholic drinks may reduce calories purchased from both types of drinks.

## INTRODUCTION

Excessive alcohol consumption is a major contributor to the global burden of non‐communicable diseases, such as cancer, heart disease and stroke [1, 2]. Interventions that alter the physical and economic environments in which alcohol‐related behaviours occur have the potential to reduce its consumption [3]. Improved labelling of alcohol products is one intervention that has been proposed, with potential to be implemented at scale [4, 5].

There is strong evidence that tobacco health warning labels (HWLs) increase a range of smoking cessation‐related behaviours [6, 7] and are a feasible population‐level intervention [8]. In addition, these effects are evident in similar magnitude among those in more and less deprived groups [9]. Evidence from online studies suggests that while both image‐and‐text HWLs—which include an often aversive visual image—and text‐only HWLs reduce hypothetical selection of alcoholic drinks, the former are more effective [10]. Initial laboratory studies suggest both are similarly effective at decreasing consumption rate [11] but that image‐and‐text HWLs may exert larger effects on abstinence and consumption intentions than text‐only HWLs [12]. There is an absence of evidence of the impact of HWLs from randomized control trials [13] as well as from studies in field settings, such as online and physical supermarkets [14, 15]. While a field study in a supermarket found alcohol sales were reduced over a 14‐month period with improved labels that included HWLs, the specific effect of the warning label could not be isolated [16]. Another study in a naturalistic shopping laboratory found no impact of HWLs upon selection or purchasing behaviour [17] but the setting lacked ecological validity, as no money was exchanged and participants did not keep the drinks they selected.

Another potential labelling intervention is the provision of calorie information, which current evidence suggests may have small effects on healthier selection and consumption of food products [18]. Many alcohol products are currently exempt from mandatory nutrition labelling, including in the UK Government’s recently implemented policy (April 2022) on calorie labelling out of the home [19]. This is despite alcohol having 7.1 kcal/g which, when compared to macronutrients, is the second highest energy value per gram after fat (9 kcal/g). Most products therefore do not display this information [20] and as a result drinkers’ knowledge of the energy content of alcoholic drinks is poor [21]. However, the UK Government’s most recent obesity strategy included plans to consult on the provision of calories on alcohol [22] and there are increasing calls for improved alcohol labelling, including through displaying calorie information [5, 23]. Current evidence on the provision of calorie information on alcohol is scarce, with a recent review finding no studies in real‐world settings or of effects on actual purchasing behaviour [24].

The principal aim of this study was to estimate the impact on selection of (a) HWLs (text‐only and image‐and‐text) on alcoholic drinks and (b) calorie labels on alcoholic and non‐alcoholic drinks. It was hypothesized that HWLs and calories labels would reduce the number of alcohol units selected.

## METHODS

The study was prospectively registered (https://www.isrctn.com/ISRCTN10313219). Both the study protocol (https://osf.io/ch2sm/) and a statistical analysis plan (https://osf.io/qwdra/) were pre‐registered on the Open Science Framework (OSF). The study was approved by the University of Cambridge ethics committee (ref: PRE.2020.155). Trial reporting follows Consolidated Standards of Reporting Trials (CONSORT) 2010 guidelines.

### Study design

The study used a parallel‐groups three [health warning labels (HWLs)]: (i) image‐and‐text HWL, (ii) text‐only HWL and (iii) no HWL) × 2 (calorie labels: present versus absent) factorial design (see Box 1).

Box 1 **Health warning label (HWL) type and calorie label displayed with the alcoholic drinks in the selection task.**

**Table:** 

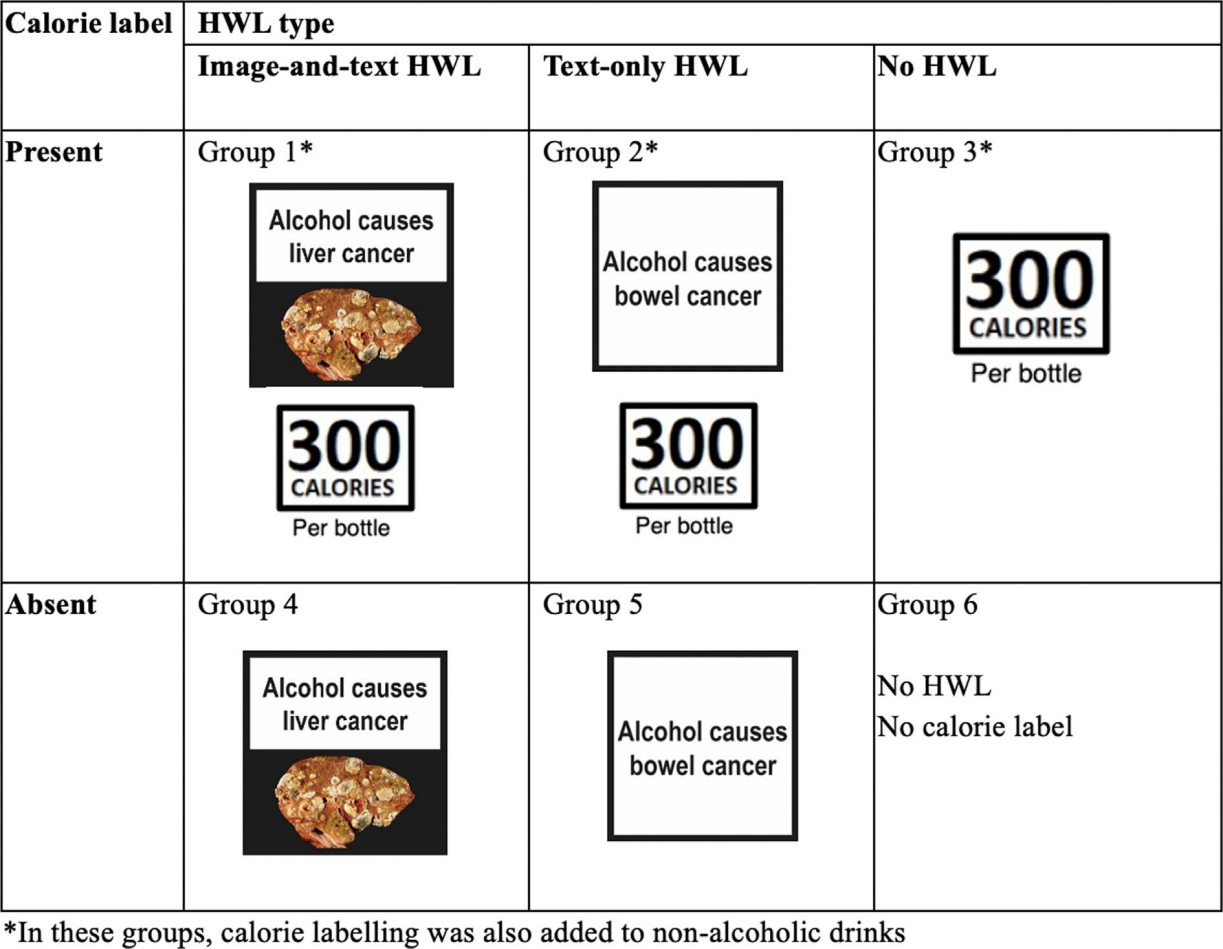

### Setting

Participants completed a simulated supermarket selection task hosted on the Qualtrics online survey platform (see Supporting information, Fig. S1a). Following this, participants were required to purchase the same drinks in Tesco on‐line supermarket (Tesco.com), the largest national supermarket in the United Kingdom.

### Participants

Eligible participants were adults (18+, the legal minimum age for alcohol use in the United Kingdom) residing in England or Wales, who self‐reported that they consumed beer or wine at least weekly and purchased these drinks at least monthly from Tesco.com, with a minimum spend of £20. Participants had to be willing to complete a shop at Tesco.com following completion of the selection task, book a delivery or click‐and‐collect slot and send proof of purchase (their receipt) to the research team. Similar proportions of males and females of a range of ages were recruited via Roots Research (https://rootsresearch.co.uk/), one of the largest research agencies in the United Kingdom, with a high‐quality panel of more than 350 000 participants. Recruitment occurred between September 2021 and March 2022.

#### Sample size

There was no direct evidence available within the literature from which to estimate the effect of the intervention on selection of multiple drink options or on the size of interaction effect for HWLs and calorie labels. A maximum sample size of 600 was possible with available resources (100 per group). An illustrative sample size calculation based on 600 participants suggests that with 85 per group (allowing for attrition of 15%) there would be 80% power and at alpha 5% (510 participants) to detect an overall interaction effect size of 0.147 or greater with a two‐way analysis of variance (ANOVA).

### Randomization and masking

Randomized assignment of participants was completed via the default algorithm in Qualtrics with a ratio of 1:1:1:1:1:1. Participants were unaware of their group assignment throughout the study. The research team were blinded to allocation until participants had completed the primary outcome and there was no possibility of contact between the research team and participants until after the primary outcome and the selection task were completed; the statistician completing the analysis was blinded to the allocation.

### Intervention

All participants viewed 64 drink options. This comprised (i) a range of beers, ciders, alcohol‐free beer and cider alternatives and soft drinks (32 options) and (ii) a range of wines, alcohol‐free wine alternatives and soft drinks (32 options), modelled on the available range of products on Tesco.com (see Supporting information, Fig. S1a). Alcoholic drinks were labelled according to the six groups in Box 1. To ensure that they were clearly visible, labels were displayed next to the product. The specific warnings—developed and tested in previous studies—were HWLs that were most effective in increasing negative emotions [25] and decreasing the odds of selecting alcohol [10]. Eight different variants of image‐and‐text HWLs and seven different variants of text‐only HWLs were used to increase variety, maximize engagement and likelihood of impact, in line with tobacco guidelines specifying which rotating warnings are most effective [26]. Illustrative examples of labelled alcohol products (Supporting information, Fig. S1b) and full details on the drink options are included in Supporting information, Fig. S1a and Table S1.

In the typology of interventions in proximal physical micro‐environments (TIPPME) [3], both health warning and calorie labelling interventions are classified as ‘information × product’ interventions.

### Outcome measures

#### Primary outcome

The primary outcome was the number of alcohol units selected in the context of a stated intention to purchase. In the United Kingdom one unit is 10 ml, or 8 g of pure alcohol. Participants were aware when selecting drinks in the task that they were required to subsequently purchase the drinks and send proof of this to the research team (otherwise they were not reimbursed). Units of alcohol were calculated for all drinks that were > 0% alcohol by volume (ABV), that is, alcoholic and ‘alcohol‐free’ drinks (defined as containing 0–0.5% ABV). This outcome was pre‐registered as the primary outcome, as it was assessed in all participants exposed to the intervention and measured within the same context; that is, the simulated online supermarket.

#### Secondary outcome measures

Secondary outcomes were the number of alcoholic and non‐alcoholic drinks selected; the number of alcohol units purchased; the proportion (i.e. percentage) of total drinks selected and purchased that were alcoholic; the total number of calories selected and purchased (overall and by drink category: alcoholic and non‐alcoholic drinks). The principal purpose of including a measure of purchasing in the actual online supermarket was to validate and strengthen our primary outcome of selection, rather than to measure purchasing behaviour in a separate context.

Additional outcomes were the total number of drinks selected and purchased and the number of alcoholic and non‐alcoholic drinks purchased.

Selection outcomes were assessed from the simulated online supermarket task and purchasing outcomes via Tesco.com receipts.

### Other measures

#### Negative emotional arousal

Assessed using a four‐item measure, previously used to assess the impact of warning labels on cigarette packages [27] and adapted for alcohol HWL studies [10, 17]. Responses were rated on seven‐point scales: ‘How (afraid/worried/uncomfortable/disgusted) does the label on this drink make you feel?’ [1, not at all (afraid/worried/uncomfortable/disgusted) to 7, very (afraid/worried/uncomfortable/disgusted)].

#### Acceptability

Assessed using one item on a seven‐point scale, adapted from previous research assessing the impact of sugar tax [28] and alcohol HWLs [10, 17]: ‘Do you support or oppose putting this label on alcoholic drinks?’ (strongly oppose–neither oppose nor support–strongly support). Ratings past the scale mid‐point, that is, more than 4, indicated that the label was acceptable.

#### Demographic characteristics

Age, gender and highest qualification attained (with classification based on UK definitions [29, 30]).

#### Household members

Number of adults (18+) and children (< 18).

#### Drinking behaviour risk

Alcohol Use Disorders Identification Test (AUDIT) [31], a 10‐item clinical screening measure for assessing risk associated with participants’ drinking behaviour (low‐risk drinking: score 0–7; medium/hazardous‐risk drinking: score 8–15; high/harmful‐risk drinking: score ≥ 16).

#### Baseline weekly unit consumption

Self‐reported drinks consumed and purchased over the previous 7 days, used to calculate the number of alcohol units as a continuous variable.

#### Manipulation check

Participants were asked if they noticed any labels on the products and to describe these, as well as what they thought the study was about.

#### Free‐text comments

Participants provided comments on the task, such as explaining their choice of drinks.

### Procedure

Participants were initially provided with an information sheet, instructions and a link to the study via e‐mail. Participants were told that the study was investigating ‘drink choices and shopping behaviour’, and were not made aware of the study aim. At the start of the study task, participants were given this information again and gave consent. Participants were randomized, and in a simulated online supermarket environment replicating Tesco.com (presented within Qualtrics) were shown the available drink selection. They chose all the drinks they wanted to purchase in their next online shop at Tesco.com, and there was no time limit within the simulated supermarket. Participants then rated the labels on negative emotional arousal and acceptability (those in the no label group were re‐randomized to a label group), before completing demographic and drinking behaviour measures.

Participants were then instructed to immediately place their selected drinks in their Tesco.com shopping basket, along with any other items, then book their delivery or collection slot, and confirm this within 48 hours. Purchases (including additional drinks) were recorded from receipts. Participants were debriefed via e‐mail and reimbursed £35(~$44) for their time taking part (but not for the drinks they purchased). For further details of the procedure see Supporting information, S1b.

### Statistical analysis

Analyses were pre‐registered in a statistical analysis plan (https://osf.io/qwdra/).

**FIGURE 1:**
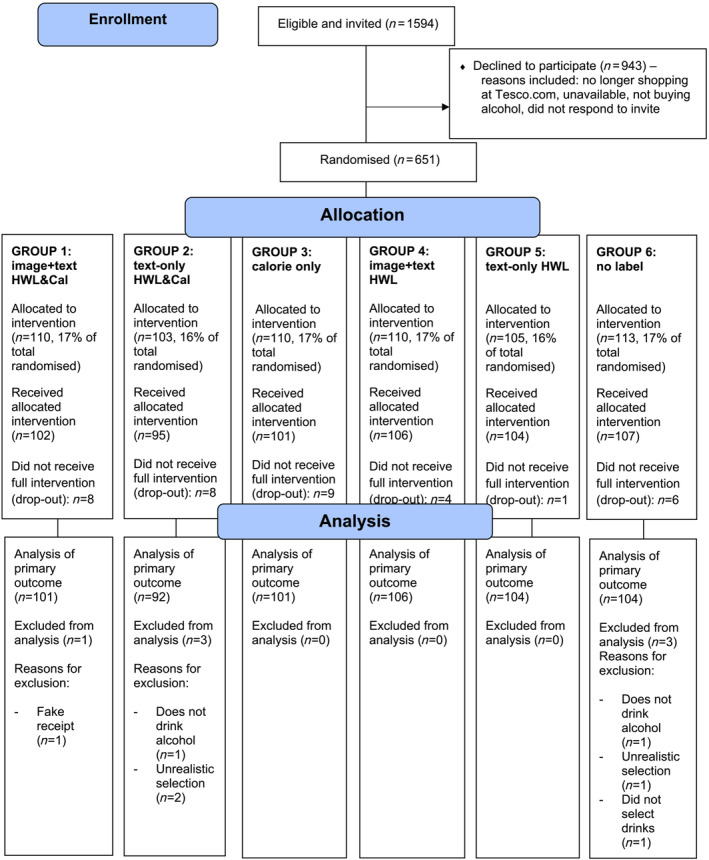
Flow of participants through study.

All participants who completed the selection task were included in the primary outcome analysis. Participants who failed to complete the selection task and those whose responses were flagged as incomplete or suspicious—for example, those who forged data (i.e. submitted fake receipts that were not generated by Tesco) or selected an unrealistically large number of drinks (e.g. more than 200 units) that were not purchased—were excluded (see Fig. 1 for details by group).

For the primary outcome a generalized linear model was used. An overall two‐way ANOVA summary and the equivalent regression summary are reported (Supporting information, S2). The model utilized the 3 × 2 design with two independent variables: (1) image‐and‐text HWL versus text‐only HWL versus no label, and (2) calorie versus no calorie labelling. Demographic (age, gender, highest qualification, ethnicity) and drinking characteristics were included as covariates in the model, but these models were only reported when conclusions were changed by their inclusion. Umbrella ANOVA *P*‐values, at a threshold for significance of 0.025 (i.e. 5%/2) are reported. The interaction terms were dropped, as there was no clear evidence of an interaction (*P* > 0.01).

For most secondary and additional outcomes, analysis of covariance (ANCOVA) and regression models were repeated as per the primary outcome. See Supporting information, S2 for full details.

Two per‐protocol analyses were pre‐specified each for the alcohol units and calories outcome. The primary outcome analysis was repeated for (i) participants who purchased what they selected, either with or without additional drinks (per‐protocol analysis 1); and (ii) only participants who purchased exactly what they selected and purchased with no additional drinks (per‐protocol analysis 2).

Free‐text comments provided by participants were manually coded and emergent themes were identified, and agreed between all authors. For full details see Supporting information, S3.

## RESULTS

### Sample characteristics

Figure 1 shows the flow of participants. In total, 651 participants were randomized (n per group 103‐113), 615 of whom completed the selection task. Six hundred and eight participants were included in the primary outcome analysis. For purchasing outcomes, of the 608 participants who completed the selection task, 467 (77%) went on to purchase drinks from Tesco.com (this was similar across groups: range = 73–80%). The primary analysis sample was 55% female and the mean age was 35.5 [standard deviation (SD) = 10.8]. Groups were well balanced on most characteristics (Table 1). The number of units consumed and purchased was lower in group 3 (calorie label only group). Weekly units purchased was therefore included as a covariate in the models. Raw primary and secondary outcome data are shown in Table 2 and model results in Table 3.

**TABLE 1:** Characteristics of participants included in the primary outcome analysis.

	Calorie label					
	Present			Absent		
	Group 1: image‐and‐text HWL	Group 2: text‐only HWL	Group 3: no HWL	Group 4: image‐and‐text HWL	Group 5: text‐only HWL	Group 6: no label
	*n* = 101	*n* = 92	*n* = 101	*n* = 106	*n* = 104	*n* = 104
Alcohol consumption previous week (units) (mean, SD)	24.86 (24.38)	23.80 (22.36)	19.03 (18.13)	25.65 (22.80)	22.31 (21.85)	29.62 (32.25)
Alcohol purchasing previous week (units) (mean, SD)	34.07 (29.25)	30.58 (27.52)	25.35 (22.72)	33.68 (27.05)	31.32 (21.78)	33.75 (28.36)
AUDIT score (mean, SD)	8.8 (4.3)	9.62 (5.37)	8.06 (4.35)	9.78 (5.32)	10.32 (5.59)	9.76 (5.52)
Low risk drinking (scores 1–7)	45 (45)	36 (40)	50 (50)	37 (35)	37 (36)	43 41)
Medium‐ to high‐risk drinking scores (8+)	56 (55)	56 (60)	51 (50)	69 (65)	67 (64)	61 (59)
Age (mean, SD)	35.64 (10.96)	33.66 (10.39)	35.53 (10.51)	35.93 (10.21)	35.43 (11.60)	36.79 (10.90)
18–39 years	28 (28)	19 (21)	26 (26)	32 (30)	30 (29)	35 (34)
40 and over	73 (72)	73 (79)	75 (74)	74 (70)	74 (71)	69 (66)
BMI (mean, SD)	26.90 (5.46)	25.71 (5.73)	25.43 (5.01)	26.65 (5.74)	26.56 (6.07)	25.92 (5.31)
Gender (*n*, %)						
Male	39 (38.6)	44 (47.8)	37 (36.6)	52 (49.1)	47 (45.2)	50 (48.1)
Female	61 (60.4)	48 (52.2)	63 (62.4)	54 (50.9)	57 (54.8)	54 (51.9)
Other	1 (1%)	0 (0)	1 (1%)	0 (0)	0 (0)	0 (0)
Household members						
Number of adults in household (mean, SD)	1.87 (0.82)	2.22 (2.11)	1.93 (0.67)	2.08 (0.70)	2.07 (0.95)	1.96 (0.72)
Number of children in household (mean, SD)	0.70 (0.97)	0.64 (0.99)	0.67 (0.98)	0.81 (0.99)	0.67 (0.92)	0.81 (1.0)
Highest qualification (*n*, %)						
No qualifications	1 (1)	0 (0)	1 (1)	2 (2)	0 (0)	0 (0)
Qualifications at level 1 and below	0 (0)	1 (1)	0 (0)	1 (1)	0 (0)	1 (1)
GCSE/O Level grade A*–C or vocational level 2 or equivalents	11 (11)	11 (12)	18 (18)	10 (9)	11 (11)	14 (13)
A levels or vocational level 3 or equivalents	19 (19)	16 (17)	17 (18)	18 (17)	21 (20)	25 (24)
Higher education or professional/vocational equivalents	69 (68)	64 (70)	64 (63)	74 (70)	71 (68)	64 (62)
Other qualification	1 (1)	0 (0)	0 (0)	1 (1)	1 (1)	0 (0)
Ethnicity (*n*, %)						
White British /white Irish/Other white background	81 (80)	67 (73)	76 (75)	86 (81)	83 (80)	83 (80)
Mixed white and black African/mixed white and Asian/mixed white and black Caribbean/Other mixed background	6 (6)	2 (3)	5 (5)	3 (3)	7 (7)	5 (5)
Asian/Asian British (Indian/Pakistani/Bangladeshi/Chinese/Other Asian/Asian British background)	9 (9)	17 (19)	12 (12)	9 (8)	5 (5)	10 (10)
Black/black British (African/Caribbean/Other black/black British background)	5 (5)	4 (4)	7 (7)	7 (7)	7 (6)	3 (3)
Other ethnic group	0 (0)	1 (1)	0 (0)	0 (0)	2 (2)	2 (2)
Prefer not to say/missing	0 (0)	(0)	1 (1)	1 (1)	0 (0)	1 (1)

Abbreviations: HWL = health warning labels; SD = standard deviation; AUDIT = Alcohol Use Disorders Identification Test; BMI = body mass indix; GCSE = general certificate gg standard education.

**TABLE 2:** Primary and secondary outcomes: raw means (± SDs), by group.

	Calorie label					
	Present			Absent		
	Group 1: image‐and‐text HWL	Group 2: text‐only HWL	Group 3: no HWL	Group 4: image‐and‐text HWL	Group 5: text‐only HWL	Group 6: no label
	*n* = 101	*n* = 92	*n* = 101	*n* = 106	*n* = 104	*n* = 104
Primary outcome						
Total alcohol units selected	25.7 (24.4)	22 (18.8)	19.6 (20.9)	21.3 (16.3)	22.0 (16)	23.7 (17.5)
Secondary outcomes: selection						
Alcohol						
Number of alcoholic drinks selected	9.38 (9.95)	7.24 (8.02)	6.58 (10.4)	7.43 (8.27)	7.61 (7.83)	7.64 (8.02)
Number of non‐alcoholic drinks selected	6.64 (9.64)	7.76 (16.5)	5.99 (9.77)	5.34 (7.28)	4.63 (6.51)	5.77 (8.23)
Proportion of alcoholic drinks selected that are alcoholic	0.64 (0.35)	0.57 (0.38)	0.55 (0.36)	0.59 (0.38)	0.63 (0.34)	0.64 (0.37)
Calories						
Total calories selected	2396 (1943)	2275 (2016)	1913 (2097)	2038 (1386)	1995 (1509)	2203 (1589)
Calories selected from non‐alcoholic drinks	492 (694)	657 (1231)	487 (768)	447 (614)	357 (530)	477 (709)
Calories selected from alcoholic drinks	1903 (1783)	1618 (1398)	1427 (1595)	1592 (1282)	1638 (1273)	1725 (1298)
Secondary outcomes: purchasing						
	*n* = 74	*n* = 71	*n* = 74	*n* = 85	*n* = 84	*n* = 79
Alcohol						
Total alcohol units purchased	25.4 (25.9)	22.8 (18.9)	17.5 (12.0)	22.8 (15.5)	22.2 (15.6)	22.9 (15.8)
Proportion of alcoholic drinks purchased that are alcoholic	0.64 (0.36)	0.59 (0.37)	0.58 (0.36)	0.64 (0.35)	0.63 (0.343)	0.62 (0.38)
Calories						
Total calories purchased	2292 (1846)	2210 (1652)	1532 (851)	2095 (1293)	1974 (1418)	2090 (1295)
Calories purchased from non‐alcoholic drinks	468 (614)	509 (685)	311 (235)	436 (646)	328 (501)	441 (664)
Calories purchased from alcoholic drinks	1874 (1748)	1701 (1408)	1221 (878)	1659 (1152)	1646 (1239)	1649 (1150)

Abbreviations: HWL = health warning labels; SD = standard deviation.

**TABLE 3:** Model results ANCOVA: *F*‐value, degrees of freedom, *P*‐values (interaction term not included as *P* > 0.01).

	HWL (overall) main effect	Calorie labelling main effect
Primary outcome		
Total alcohol units selected	*F*_(2,599)_ = 0.406, *P* = 0.666	*F*_(1,599)_ = 0.002, *P* = 0.961
Secondary outcomes: selection		
Alcohol		
Number of alcoholic drinks selected	*F*_(2,599)_ = 1.042, *P* = 0.354	*F*_(1,599)_ = 0.063, *P* = 0.802
Number of non‐alcoholic drinks selected	*F*_(2,599)_ = 0.016, *P* = 0.984	*F*_(1,599)_ = 3.482, *P* = 0.063
Calories		
Total calories selected	–	*F*_(1,599)_ = 0.624, *P* = 0.430
Calories selected from alcoholic drinks	–	*F*_(1,599)_ = 0.001, *P* = 0.980
Calories selected from non‐alcoholic drinks	–	*F*_(1,599)_ = 3.23, *P* = 0.072
Secondary outcomes: purchasing		
Alcohol		
Total alcohol units purchased	*F*_(2,462)_ = 1.85, *P* = 0.159	*F*_(1,462)_ = 0.193, *P* = 0.661
Calories		
Total calories purchased	–	*F*_(1,462)_ = 0.089, *P* = 0.766
Calories purchased from alcoholic drinks	–	*F*_(1,462)_ = 0.328, *P* = 0.567
Calories purchased from non‐alcoholic drinks	–	*F*_(1,462)_ = 0.262, *P* = 0.609

*Note*: Beta‐binomial regression models were used for proportion outcomes, these are reported in the regression table, together with the other regression results (Supporting information, S2).

Abbreviations: ANCOVA = analysis of covariance; HWL = health warning labels.

### Primary outcome

There was no evidence of an overall difference for (a) HWLs or (b) calorie labels on the number of alcohol units selected (HWLs: *F*_(2,599)_ = 0.406, *P* = 0.666; calorie labels: *F*_(1,599)_ = 0.002, *P* = 0.961).

### Secondary outcomes

#### Alcohol selected and purchased

There was no evidence of an overall difference for alcohol selection or purchasing on any of the secondary outcomes, including the number of alcohol units purchased (*P*s > 0.06).

#### Calories selected and purchased

There was no evidence of an effect of calorie labels on total calories selected or total calories purchased (*P*s > 0.07).

#### Subgroup analyses of ‘calorie label only’ group versus ‘no label’ group

These pre‐specified analyses compared the effect on calories selected and purchased of those randomized to the ‘calorie label only’ group (group 3; *n* = 101) with those randomized to the ‘no label’ group (group 6; *n* = 104) (i.e. this analysis included 205 of 608 randomized participants).

There was no evidence of a difference in calories selected between the ‘calorie label only’ and the ‘no label’ groups (*P* = 0.643).

Amongst the 75% (153 of 205 participants) in these two groups who went on to purchase drinks (calorie label only: 74 of 101; no label: 79 of 104), fewer calories were purchased by those in the calorie label only group [*P* = 0.0282, 20% reduction, 95% confidence interval (CI) = −35%, −2%]. When examined separately by category (using different models), this effect was evident both for alcoholic (*P* = 0.0229, 22% reduction, 95% CI = −37%, −3%) and for non‐alcoholic drinks (*P* = 0.0086, 34% reduction, 95% CI = −51%, −10%) (Supporting information, S2).

To explore the difference in findings between calorie selection and purchasing, demographic and drinking characteristics of participants who did not go on to purchase (*n* = 141) were compared to those who purchased the drinks they selected (*n* = 467). Purchasers tended to be more educated and older, and self‐reported purchasing and consuming less alcohol (Supporting information, S4).

### Per‐protocol analyses (Table 4)

**TABLE 4:** Per‐protocol analyses: model results ANCOVA: *F*‐value, degrees of freedom, *P*‐values.

	HWL (overall) main effect	Calorie labelling main effect
Alcohol selection		
Per‐protocol analysis 1: number of alcohol units selected (exact match, with or without additional drinks) (*n* = 456)	*F*_(2,451)_ = 0.793, *P* = 0.453	*F*_(1,451)_ = 1.059, *P* = 0.304
Per‐protocol analysis 2: number of alcohol units selected (exact match with no additional drinks) (*n* = 426)	*F*_(2,422)_ = 0.920, *P* = 0.399	*F*_(1,422)_ = 0.687, *P* = 0.408
Calorie selection		
Per‐protocol analysis 1: total calories selected (including all additional drinks) (*n* = 456)	–	*F*_(1,451)_ = 0.804, *P* = 0.370
Per‐protocol analysis 2: total calories selected (exact match with no additional drinks) (*n* = 426)	–	*F*_(1,422)_ = 0.417, *P* = 0.519

Abbreviations: ANCOVA = analysis of covariance; HWL = health warning labels.

#### Alcohol selected

There was no evidence for an overall difference of (a) HWLs or (b) calorie labels on the number of alcohol units selected in those who purchased the drinks they selected, either with or without additional drinks (*P*s > 0.3).

#### Calories selected

There was no evidence for an overall difference of calorie labels on the number of calories selected in those who purchased the drinks they selected, either with or without additional drinks (*P*s > 0.3).

There was a reduction in number of calories selected in the ‘calorie label only’ group (group 3) compared to the ‘no label’ group (group 6) in those who purchased the drinks they selected (*n* = 205 of 608 randomized), both with additional drinks (*P* = 0.024, 19% reduction, 95% CI = −34%, −1%) and without (*P* = 0.03, 20% reduction, 95% CI = −35%, −2%).

### Additional outcomes

There was evidence to suggest image‐and‐text and text‐only HWLs increased the total number of alcoholic drinks purchased (image‐and‐text HWL: 42% increase, 95% CI = 5%, 80%, *P* = 0.03); text‐only HWL: 39% increase, 95% CI = 1%, 76%, *P* = 0.046). There was no evidence for an overall difference of (a) HWLs or (b) calorie labels on any other additional outcomes (Supporting information, S5).

#### Label ratings and manipulation check

Based on comparing means and 95% CIs, calorie labels were rated as more acceptable and had lower scores for negative emotional arousal (i.e. lower fear, disgust, worry, discomfort) than all image‐and‐text and text‐only HWLs (Supporting information, S6).

In the labelling groups, 74% (370 of 504) of participants indicated that they noticed the labels, of whom a clear majority correctly described them (357 of 370).

### Analysis of free‐text comments

Four hundred general comments were left at the end of the study that contained content suitable for analysis. Responses to HWLs have been qualitatively analysed in previous research [17, 25], but calorie labels have received less attention. Box 2 therefore outlines the main themes that emerged from responses to calorie labels.

Box 2 **Calorie specific comments.^a^**

**Table:** 

Theme	Theme description	Example comments
Healthfulness	Calorie information can be helpful for making healthier choices	‘I felt more drawn to the alcohol‐free drinks and will be trying to buy these more as I like to look after my body and diet and seeing the calorie content also made me think that they are a better option’ ‘I think the labels are a great idea. People are becoming more health conscious so being able to see how many calories are in drinks will help us make healthier choices’
Switching to lower calorie options	Drink comparison based on calorie content and selection of lower calorie options	‘The calories next to the item was useful, as you do start to compare items based on the lowest number’ ‘It’s interesting to see that e.g. the alcohol‐free beer is only 20 calories and that a glass of low‐alcohol wine is less than half the calories of a normal glass’
Comparing calorie labels with HWLs	Comparison with HWLs: calorie information as more acceptable or effective option	‘I'm happy to see the calorie content highlighted, if necessary. However, would prefer not to see sad images on something that equals fun time in my life. I am aware of risks involved, eat very healthily, participate in a lot of sport, but feel the images on the bottles are not the right platform to educate people’ ‘It’s a good idea to provide calorie information clearly on labels, that would influence my drink choice rather than warnings about liver disease’
Hidden calories	Lack of awareness/underestimation of ‘hidden’ calories in alcoholic drinks	‘I think the calorie count is a very good idea as people are mostly unaware of the secret calorie count of alcohol’ ‘I was not aware of the calories in alcohol and I do watch calories so it may make me think more carefully about what kind of alcohol I drink’ ‘It is good to advertise the hidden calories’
Potential adverse effects	Calorie information could have adverse effects in those with eating disorders	‘I would appreciate this as I am currently calorie counting, however I can imagine this may be triggering for people with eating disorders’

^a^ A detailed overview of all themes can be found in Supporting information, S3.

### Data sharing statement

Data is available from the Open Science Framework (https://osf.io/4xfw5) and the University of Cambridge Research Repository.

## DISCUSSION

We found no evidence that either health warning labels—image‐and‐text or text‐only—describing the adverse effects of alcohol consumption, or calorie labels, changed the number of alcohol units selected or purchased in an online purchasing setting.

In pre‐specified subgroup analyses comparing the ‘calorie label only’ group to the ‘no label’ group, there was no effect on calorie selection. Of those who went on to purchase drinks there was some evidence suggesting that calorie labels on alcoholic and non‐alcoholic drinks might reduce calories purchased from both types of drinks.

### Interpretation of findings

The null findings for the impact of HWLs on all selection and purchasing outcomes were contrary to predictions, although these predictions were made in the context of extremely limited pre‐existing evidence. The findings accord with our previous experimental study in a naturalistic shopping setting, where the same image‐and‐text and text‐only HWLs had no impact on selection [17]. Previous studies that have reported positive effects of alcohol HWLs on selection or purchasing behaviour have predominantly been conducted in online hypothetical settings [4, 10, 14, 32], suggesting that differences in findings may be explained by the study setting and/or nature of the outcome measure [15]. These findings are not in line with tobacco or food research, which suggests that HWLs can change behaviour [33, 34]. However, caution should be applied when comparing findings from food or tobacco studies to alcohol as they are different products, and smoking and eating behaviours differ in important respects to that concerning alcohol [35]. It may therefore be that alcohol HWLs—in either image‐and‐text or text‐only form—are not sufficient to change real‐world purchasing behaviour. The provision of additional information alongside health harms, such as drinking guidelines, could increase their potential impact [36]. Alternatively, it may be that short‐term exposure to HWLs on a single shopping occasion is insufficient, but effects are elicited over a longer‐term period with repeated exposure [37] or there are effects on consumption. For example, HWLs increase awareness of health harms which may lead to behaviour change [38, 39]. However, awareness is neither necessary nor sufficient for behaviour change, and it is also possible that HWLs could work via non‐conscious routes [40] such as via low‐level associative mechanisms affecting implicit motivations [41, 42]. The only other study that we are aware of that included actual (i.e. not hypothetical) behavioural outcomes found that labels that included text‐only health warnings reduced alcohol sales over a 14‐month period [16], suggesting potential longer‐term effects. However, this study could not isolate the specific impact of the warning label as there were multiple labels in rotation and the warnings were halted after 1 month due to industry backlash [43].

In a planned analysis comparing only two groups there was no evidence for an effect of calorie labelling on alcohol selection. In those participants who went on to purchase drinks, fewer calories were purchased from both alcoholic and non‐alcoholic drinks. Although these results suggest that calorie labelling may have the potential to impact purchasing behaviour due caution should be applied, given that they were from a subgroup analysis with a relatively small sample size. Furthermore, exploratory analyses suggested that in the whole sample, the 77% of participants who went on to purchase differed in their demographic and drinking characteristics—they tended to be older, more educated and self‐reported drinking less alcohol. The only comparable evidence to date on calorie purchasing comes predominantly from food and soft drink studies where a Cochrane Review—currently being updated [44]—identified limited evidence suggesting small effects on purchasing, but with considerable uncertainty [18]. There was no evidence for an effect of calorie labels when they were combined with health warning labels. This could be explained by an information overload effect, which posits that too much information on products can overload cognitive capacity and impair the quality of decisions [45]. Alternatively, it could simply be that HWLs distracted participants from the calorie label.

In terms of acceptability, which is a key factor in the likelihood that an intervention will be implemented [46], calorie labels were rated as acceptable, and more so than either type of HWL. This accords with the majority of free‐text comments being coded as positive, as well as other evidence of public support for alcohol calorie labelling [47]. Calorie labels were also rated lower on negative emotional arousal. Text‐only HWLs were rated as higher on acceptability and lower on negative emotional arousal than image‐and‐text HWLs, consistent with previous studies [10, 17, 25]. As negative emotional arousal has been highlighted as an important component of the underlying mechanism of the effect of health warnings [10], it should be included in future studies designed to directly test the causal pathways for labelling interventions, along with other potential mediators.

Free‐text comments indicated that calorie information was perceived as a helpful intervention to encourage healthier choices through switching to lower calorie options, as well as highlighting the calories contained in alcohol, of which many people are unaware [24]. However, previous research has also suggested that calorie labelling could encourage compensatory behaviours with the potential to harm health, such as reducing food intake or selecting drinks with fewer calories but greater alcohol content, to maximize alcohol intake while minimizing energy intake [48]. The possibility of harmful unintended consequences of calorie labelling warrant further study. Attitudes towards HWLs in the current study were similar to those observed in previous studies, with negative emotional reactions—including shock, disgust and fear—being common, and mixed views concerning their potential effectiveness and acceptability [17, 25].

### Strengths and limitations

To our knowledge, this is the first randomized controlled trial to estimate the impact of HWLs and calorie labelling on drinks in a naturalistic setting. Meaningful selection and actual purchasing outcomes were assessed, with participants able to complete their typical online shop, including selecting and purchasing freely from a wide range of drinks. Additionally, it provides proof of concept for further research using a similar simulated shopping paradigm that incorporates actual purchasing.

The study also had some limitations. First, while the primary selection outcome was assessed with a stated intention to purchase, subsequent purchasing was not mandated or forced and there was substantial dropout (23%) between selection and actual purchasing. Although this study required participants to transfer between simulated and actual supermarkets which may have exacerbated the degree of attrition, some dropout may be inevitable when assessing behaviour in online shopping contexts, given the inevitable time gaps between selecting and ultimately purchasing products. For example, ‘cart abandonment’—where people do not purchase items they put in their shopping cart—is common in online (including supermarket) shopping contexts [49]. Retention to the point of actual purchasing was also significantly improved (from 66% to 77%) relative to our previous study using a similar protocol [50], probably explained by an increased financial incentive and the refinement of study instructions. Additionally, the majority of participants who purchased also went on to purchase the exact drinks they selected, indicating that this study procedure is feasible and effective in measuring objective selection and purchasing in online shopping settings; future studies using a similar method should account for a similar degree of attrition.

Secondly, participants in the current study sample were of a higher socio‐economic status than the UK average [51], although this is probably representative of those who regularly purchase online at Tesco [52] and who consume alcohol [53]. As discussed in the ‘Interpretation of findings’ section, exploratory analyses suggested that participants who went on to purchase drinks for whom calorie labels reduced purchasing differed in their demographic and drinking characteristics. Given that previous research suggests that certain groups are more likely to use calorie information [54], further studies to determine the probable impact of calorie labels in a wider range of populations and population subgroups are required, with heavier drinkers being a particularly important focus. Differences between selection and purchasing in the current study could also be explained by participants who dropped out before purchasing never having been intending to purchase, and/or being less engaged with the study.

Thirdly, the sample size was determined based on available resource, and therefore it may be that some effects were smaller than the study was powered to detect. For example, there were four fewer alcohol units selected in the calorie label group compared to the no label group, and in the planned subgroup analyses, reductions in calories selected of −5% to −10% were observed. These effects were not statistically significant, but could represent potentially meaningful reductions from a population health perspective. Future studies should be suitably powered to detect smaller effects.

### Implications for future research and policy

This study suggests that short‐term exposure to HWLs may not be sufficient to change purchasing behaviour. The impact of longer‐term or repeated exposure is unknown and merits investigation.

Calorie labels show promise and warrant further evaluation, particularly given current government interest in their potential implementation in the United Kingdom [19, 22] and internationally: for example, Ireland recently passed legislation that requires energy content information on alcohol packaging [55]. The World Health Organization recommends that successful alcohol labelling legislation should include information about the harm from alcohol [56] and be consistent with non‐alcoholic drink labelling, including the provision of calorie information [55]. Regardless of whether or not labelling can elicit meaningful effects on behaviour, information on calories can enable people to accurately estimate calorie intake from drinks [57] and appears to be highly acceptable to the public. It may also lead to indirect impacts, for example by encouraging industry and supermarkets to increase the availability or promotion of lower calorie alternatives [44, 58, 59].

### CONCLUSIONS

There was no evidence that health warning labels reduced the number of alcohol units selected or purchased in an online retail context. There was some evidence suggesting that calorie labels on alcoholic and non‐alcoholic drinks may reduce calories purchased from both types of drinks. Given that this is the first study to date assessing the impact of calorie labels on alcohol selection and actual purchasing, considerable caution is needed in interpreting these findings. Further evaluation is warranted in suitably powered studies in real‐world settings.

## AUTHOR CONTRIBUTIONS

**Natasha Charlotte Clarke:** Conceptualization (equal); data curation (equal); methodology (equal); project administration (lead); writing—original draft (lead); writing—review and editing (lead). **Jennifer Ferrar:** Data curation (equal); project administration (equal); writing—review and editing (equal). **Emily Pechey:** Data curation (equal); project administration (equal); writing—review and editing (equal). **Minna Ventsel:** Data curation (equal); project administration (equal); writing—review and editing (equal). **Mark Pilling:** Data curation (equal); formal analysis (lead); writing—original draft (equal); writing—review and editing (equal). **Marcus Munafo:** Conceptualization (equal); funding acquisition (equal); methodology (equal); writing—review and editing (equal). **Theresa M. Marteau:** Conceptualization (equal); funding acquisition (equal); methodology (equal); supervision (equal); writing—review and editing (equal). **Gareth J. Hollands:** Conceptualization (equal); funding acquisition (equal); methodology (equal); supervision (equal); writing—original draft (supporting); writing—review and editing (equal).

## DECLARATION OF INTERESTS

None to declare.

## TRIAL REGISTRATION

Pre‐registered protocol (https://osf.io/ch2sm/) and prospective ISCRTN registration: https://www.isrctn.com/ISRCTN10313219.

## Supporting information


**Supporting Information S1.** Drink options, study procedure details and example images
**Supporting Information S2.** Model results for primary and secondary outcomes
**Supporting Information S3.** Free‐text comment analysis
**Supporting Information S4.** Demographic characteristics: exploratory analysis
**Supporting Information S5.** Additional outcomes
**Supporting Information S6.** Label ratings

## Data Availability

Data is available on the Open Science Framework (https://osf.io/4xfw5, together with the study protocol and statistical analysis plan) and the University of Cambridge Research Repository.
